# Fatty acid synthase (FASN) regulates the mitochondrial priming of cancer cells

**DOI:** 10.1038/s41419-021-04262-x

**Published:** 2021-10-21

**Authors:** Barbara Schroeder, Travis Vander Steen, Ingrid Espinoza, Chandra M. Kurapaty Venkatapoorna, Zeng Hu, Fernando Martín Silva, Kevin Regan, Elisabet Cuyàs, X. Wei Meng, Sara Verdura, Aina Arbusà, Paula A. Schneider, Karen S. Flatten, George Kemble, Joan Montero, Scott H. Kaufmann, Javier A. Menendez, Ruth Lupu

**Affiliations:** 1 Division of Experimental Pathology, Department of Laboratory Medicine and Pathology, Mayo Clinic, Rochester, MN 55905 USA; 2 Mayo Clinic Cancer Center, Rochester, MN 55905 USA; 3 Department of Preventive Medicine, John D. Bower School of Population Health, University of Mississippi Medical Center, Jackson, MS 39216 USA; 4 Cancer Institute, School of Medicine, University of Mississippi Medical Center, Jackson, MS 39216 USA; 5 Institute for Bioengineering of Catalonia (IBEC), The Barcelona Institute of Science and Technology (BIST), 08028 Barcelona, Spain; 6 Department of Experimental Pathology, Mayo Clinic, Rochester, MN 55905 USA; 7 Girona Biomedical Research Institute, 17190 Salt Girona, Spain; 8 Program Against Cancer Therapeutic Resistance (ProCURE), Metabolism & Cancer Group, Catalan Institute of Oncology, 17007 Girona, Spain; 9 Deparment of Oncology, Mayo Clinic, Rochester, MN 55905 USA; 10 Sagimet Biosciences (formerly 3-V Biosciences), San Mateo, CA 94402 USA; 11 Department of Biochemistry and Molecular Biology Laboratory, Mayo Clinic Laboratory, Rochester, MN 55905 USA; 12 Present Address: Helmholtz Pioneer Campus, Heimholtz Zentrum München, Deutsches Forschungszentrum für Gesundheit und Umwelt (GmbH), Ingolstädter Landstraße 1 D-85764 Neuherberg, Munich, Germany; 13 Present Address: Department of Nutrition, Dietetics, and Hospital Management, Auburn University, Auburn, AL 36849 USA; 14 Present Address: Radiation Oncology Research, Mayo Clinic, Rochester, MN 55905 USA

**Keywords:** Cancer metabolism, Lipid signalling

## Abstract

Inhibitors of the lipogenic enzyme fatty acid synthase (FASN) have attracted much attention in the last decade as potential targeted cancer therapies. However, little is known about the molecular determinants of cancer cell sensitivity to FASN inhibitors (FASNis), which is a major roadblock to their therapeutic application. Here, we find that pharmacological starvation of endogenously produced FAs is a previously unrecognized metabolic stress that heightens mitochondrial apoptotic priming and favors cell death induction by BH3 mimetic inhibitors. Evaluation of the death decision circuits controlled by the BCL-2 family of proteins revealed that FASN inhibition is accompanied by the upregulation of the pro-death BH3-only proteins BIM, PUMA, and NOXA. Cell death triggered by FASN inhibition, which causally involves a palmitate/NADPH-related redox imbalance, is markedly diminished by concurrent loss of BIM or PUMA, suggesting that FASN activity controls cancer cell survival by fine-tuning the BH3 only proteins-dependent mitochondrial threshold for apoptosis. FASN inhibition results in a heightened mitochondrial apoptosis priming, shifting cells toward a primed-for-death state “addicted” to the anti-apoptotic protein BCL-2. Accordingly, co-administration of a FASNi synergistically augments the apoptosis-inducing activity of the dual BCL-X_L_/BCL-2 inhibitor ABT-263 (navitoclax) and the BCL-2 specific BH3-mimetic ABT-199 (venetoclax). FASN inhibition, however, fails to sensitize breast cancer cells to MCL-1- and BCL-X_L_-selective inhibitors such as S63845 and A1331852. A human breast cancer xenograft model evidenced that oral administration of the only clinically available FASNi drastically sensitizes FASN-addicted breast tumors to ineffective single-agents navitoclax and venetoclax in vivo. In summary, a novel FASN-driven facet of the mitochondrial priming mechanistically links the redox-buffering mechanism of FASN activity to the intrinsic apoptotic threshold in breast cancer cells. Combining next-generation FASNis with BCL-2-specific BH3 mimetics that directly activate the apoptotic machinery might generate more potent and longer-lasting antitumor responses in a clinical setting.

## Introduction

Elevated de novo fatty acid (FA) biogenesis driven by the activation of lipogenic enzymes is one of the most common metabolic traits that provide proliferative and survival advantages to tumors [1–7]. Fatty acid synthase (FASN) is a key enzyme in the endogenous lipogenesis pathway that primarily catalyzes the synthesis of the long-chain saturated FA palmitate from acetyl-CoA and malonyl-CoA, using NADPH as a reducing agent [8–11]. FASN activation is an early and near universal hallmark of most human carcinomas and their precursor lesions, and is enhanced in a stage-dependent manner that associates with worsened patient survival and therapeutic resistance. Interest in FASN-driven lipid signaling as a target for therapeutic intervention stemmed from findings more than a decade ago that tumor cells show reduced growth and viability upon targeted FASN suppression [8, 9, 11–14]. Since then, however, we have been unable to resolve the discrepancy between the basic science-discovery *bench* aspects of FASN blockade and the awaited *bedside* effects of FASN inhibitors (FASNis).

The demonstration of target engagement and early signs of clinical activity in cancer patients receiving next-generation FASNis—TVB-2640 [11]—has reignited interest in FASN as a target for new drug development. Unfortunately, the molecular determinants of cancer cell sensitivity to FASNis are unclear, mostly because the biological mechanisms responsible for FASNis-induced cell death are largely unknown [8–14]. Cancer cells can satisfy their demand for FAs by active uptake from the bloodstream [15], and the requirement of FASN in malignant transformation would be unrelated to its capacity to cell-autonomously generate endogenous lipids [16]. FASN activity accounts for the highest cell consumption of not only NADPH but also acetyl-CoA [8], thereby unlocking IDH1-dependent reductive carboxylation to ensure the production of reduced equivalents to counterbalance the mitochondrial oxidative stress and overcome anoikis [17–20]. Whether the essentiality of FASN to generate the necessary reductive power to quench an excessive production of reactive oxygen species (ROS) might similarly explain why FASNis ultimately determine if the mitochondrial apoptotic pathway is activated or not [21–25] remains unexplored.

**Fig. 1:**
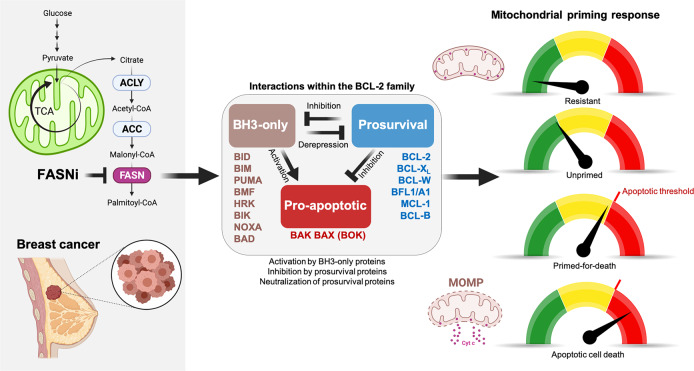
FASN and the cell death decision circuitry controlled by the BCL-2 family. We envisioned that the functional interaction between FASN-catalyzed endogenous fatty acid biogenesis and the BCL-2 family interaction network that controls the mitochondrial pathway of apoptosis might be the basis for the differential sensitivity to FASN inhibitors (FASNis). The BCL-2 family can be divided into three classes, namely the pro-apoptotic BAX/BAK proteins, the proapoptotic BH3-only proteins, and the pro-survival proteins, the latter inhibiting the activity of the pro-apoptotic BCL-2 family members. Although historically those BH3-only proteins able to directly activate BAX/BAK have been termed either “activators” and those targeting pro-survival proteins to indirectly activate BAX/BAK have been called “sensitizers”, this strict categorization is no longer appropriate as some “sensitizers” can exhibit direct activation functions under certain circumstances. Thus, BH3-only proteins can be better distinguished by their ability to either directly bind and “activate” BAX/BAK or indirectly “derepress” pro-survival proteins (via hindering the ability of pro-survival proteins to sequester BH3-only proteins to stop them activating BAX/BAK or impeding the ability of pro-survival proteins to bind to activated BAX/BAK and prevent their homo-oligomerization). BAX/BAK activation ultimately controls mitochondrial outer membrane permeabilization (MOMP), which precedes the release of mitochondria-stored cytochrome c (Cyt c) into the cytosol to promote apoptosome formation, subsequent activation of effector caspases, and apoptosis. The balanced interactions within the BCL-2 family involving activation of BH3-only proteins and inhibition by pro-survival proteins lastly sets how close a cell is to the threshold or apoptosis, a property called “mitochondrial priming”. Lack or low levels of activating/de-repressing events upon exposure to FASNis might lead to a resistant or unprimed state. When FASN blockade drives a high mitochondrial priming, cancer cells might acquire a primed-for-death state in which they ensure their survival by becoming “addicted” to anti-apoptotic proteins sequestering pro-apoptotic members. Created with BioRender.com.

Here, we hypothesized that the intrinsic variability of cancer cells to promote mitochondrial oxidative stress and engage the death decision circuitry controlled by BCL-2 family interactions centrally contributes to the response to FASNis (Fig. 1). We now uncover a novel FASN-driven facet of the so-called “mitochondrial priming” that mechanistically links the redox-buffering mechanism of FASN activity to the intrinsic apoptotic threshold in cancer cells. We demonstrate that cancer cells treated with FASNis can acquire a “primed-for-death” mitochondrial state with apoptotic hypersensitivity to BCL-2 specific BH3-mimetics. The discovery that starvation of endogenously produced FAs is a metabolic stress that heightens mitochondrial apoptotic priming might open a new avenue to rationally use next-generation FASNis and BH3 mimetic drugs for combinatorial optimization in cancer therapy.

## Materials and methods

### Cell lines

The breast cancer cell lines BT-474, MDA-MB-231, and MCF-7 were obtained from the ATCC and were maintained in IMEM (Cellgro, Mediatech Inc., Manassas, VA) supplemented with 5% fetal bovine serum (FBS, Atlanta Biologicals, Lawrenceville, GA) at 37°C in a humidified 5% CO_2_ atmosphere. Cells were authenticated to ensure their identity using a short tandem repeat profiling method provided by the Genotyping Shared Resource at Mayo Clinic Rochester. HAP1 and HAP1 *FASN-KO* cells (#HZGHC003700c006) were obtained from Horizon Discovery Ltd. (Cambridge, UK) and maintained at 37^o^C with 5% CO_2_ in IMDM medium (Gibco) supplemented with 10% FBS, 2 mmol/L L-glutamine, and 100 IU/mL penicillin/streptomycin. Cells were tested to confirm the absence of mycoplasma using the MycoAlert^®^ Mycoplasma Detection Kit (Lonza, Walkersville, MD).

### Reagents

Reagents were purchased from the following suppliers: C75 (#2489) from Tocris Bioscience (Minneapolis, MN); ABT-263/navitoclax (#A3007) from APExBio (Houston, TX); ABT-199/venetoclax (#CT-A199) from ChemieTek (Indianapolis, IN); TVB-3166 (#SML1694) from Sigma-Aldrich (St. Louis, MO); S63845 (#HY-100741) and A1331852 (#HY-19741) from MedChemExpress (Monmouth Junction, NJ); TVB-3664 was provided by 3V-Biosciences/Sagimet Biosciences (San Mateo, CA); allophycocyanin (APC)-conjugated annexin V and binding buffer from BD Biosciences (San Diego, CA); CHAPS, propidium iodide, dimethyl pimelimidate, leupeptin, pepstatin, microcystin, sodium vanadate, NaF, NAC, cerulenin and protein A-Sepharose from Sigma-Aldrich (St. Louis, MO). The p38 inhibitor SB203580 (#5633) and the JNK inhibitor SP600125 (#42-011-925MG) were purchased from Cell Signaling Technology Inc. (Danvers, MA) and Fisher Healthcare (Waltham, MA), respectively. Anti-BAK (#12105), anti-BAX (#2772), anti-BCL-2 (#2872), anti-BCL-X_L_ (#2762), anti-MCL-1 (#45725), and anti-BIM (#2819) antibodies were purchased from Cell Signaling Technology, Inc. (Danvers, MA). Anti-FASN antibody (#610963) was purchased from BD Transduction Laboratories™/BD Biosciences (San Jose, CA). Anti-NOXA antibody (#114C307, #OP180) was purchased from Merck/Sigma-Aldrich. Anti-PUMA (#28226) and β-actin (#1615 and #66009) were purchased from Santa Cruz Biotechnology, Inc. (Santa Cruz, CA).

### Constitution of palmitate-bovine serum albumin (BSA) and BSA-C75 complexes

Palmitate (Sigma-Aldrich) was complexed with BSA. Palmitate was dissolved in ethanol to 150 mmol/L and diluted 1:5 in a 4% (w/v) BSA solution in 0.9% NaCl and incubated for 1 h at 37°C to obtain a 30 mmol/L stock of BSA-complexed palmitate. C75 was then incubated with different concentrations of palmitate-BSA complex for 9 h at 37°C and used for experiments.

### Annexin V binding by flow cytometry

Binding of APC-conjugated annexin V to cells was assessed by flow cytometry. 20,000 events of pooled adherent and floating cells were collected from the FL3 (excitation 488 nm, emission 650 LP) and FL4 (excitation 635 nm, emission 661 ± 8 nm) channels of a Becton Dickinson Accuri C6 flow cytometer, and data were analyzed using Accuri C6Flow software.

### Assessment of intracellular ROS

Intracellular ROS production was monitored using the permeable fluorescence dyes 5-(and-6)-carboxy-2,7-dichlorodihydrofluorescein diacetate (carboxy-H_2_DCFDA) (Invitrogen) and dihydroethidine (DHE) (Sigma-Aldrich). Fluorescence intensity of intracellular DCF (excitation 488 nm, emission 530 nm) or ethidium (excitation 488 nm, emission 585 nm) was measured and analyzed by flow cytometry using Accuri C6Flow software.

### Mitochondrial membrane potential

Changes in ∆ψ_m_ were estimated using a flow cytometer-based analysis method for the JC-1 probe (Molecular Probes protocol: MitoProbe JC-1 assay kit for flow cytometry).

### Cytochrome c release assay

Cytochrome c release was assayed using the InnoCyte^TM^ Flow Cytometry Cytochrome c Release Kit (Calbiochem, Los Angeles, CA).

### Determination of NADPH/NADP^+^ ratio

The NADPH/NADP^+^ ratio was assessed using an NADP^+^/NADPH quantification kit (BioVision, Mountain View, CA).

### Western blot analysis

Cells were harvested and lysed in 1× cell lysis buffer (Cell Signaling Technology) containing protease and phosphatase inhibitors (Roche, Indianapolis, IN) on ice for 30 min with recurrent mixing every 5 m. Protein concentrations were determined using the Pierce BCA^®^ protein assay kit (Pierce, Rockford, IL). Cell lysates aliquots were resolved on 4–15% polyacrylamide gels (Criterion TGX precast Gel; Bio-Rad, Hercules, CA) and transferred to PVDF membranes. Membranes were blocked with 5% BSA for 1 h at RT and incubated with primary antibodies overnight at 4°C. Membranes were washed 3 times with TBS-T, incubated with horseradish peroxidase-linked secondary antibodies (1:4,000) for 1 h at RT, and visualized using enhanced chemoluminescence reagent (Pierce) and Hyperfilm.

### Quantitative reverse transcriptase-polymerase chain reaction

RNA was isolated using the Qiagen RNEasy plus mini kit. Analysis was performed in triplicate using RNA (100 ng) and TaqMan One-Step RT-PCR Master Mix (Applied Biosystems, Carlsbad, CA). Probe sets were: *Noxa* (Hs00560402_m1), *Bim* (Hs00197982_m1), *Puma* (Hs00248075_m1), *Bid* (Hs00609632_m1), *Bik* (Hs00154189_m1), *Bax* (Hs00180269_m1), *Bak* (Hs00832876_g1), *Bcl-2* (Hs00608023_m1), *Bcl-x*_*L*_ (Hs00236329_m1), and *Mcl-1* (Hs03043898_m1). PCR was performed on the ABI Prism 7900HT real-time system using a program consisting of 48°C for 30 min, 95°C for 10 min, then 40 cycles of 95°C for 15 s and 60°C for 1 min. Data analyses were performed using the following equations: Δ*C*_t_ = *C*_t_(sample) − *C*_t_(endogenous control); ΔΔ*C*_t_ = Δ*C*_t_(sample) − ΔC_t_(untreated); and fold change = 2-ΔΔ*C*_t_.

### Small interfering RNA (siRNA) transfection

RNA interference was performed using siRNAs directed against: *Noxa* (5′-GGAGAUUUGGAGACAAACU-3′), *Puma* (5′-GCCUGUAAGAUACUGUAUA-3′), or *Bim* (5′-GACCGAGAAGGUAGACAAU-3′), all from Ambion (Austin, TX). On day one, 1 × 10^7^ cells were suspended in 370 μL of medium containing 1 mmol/L oligonucleotides and subjected to electroporation using a BTX 830 square wave electroporator (BTX, San Diego, CA) delivering a single pulse at 200 mV for 10 m. Twenty-four hours after transfection, cells were treated with diluent or C75 for an additional 48 h and then assayed for annexin V binding by flow cytometry.

### Dynamic BH3 profiling

Dynamic BH3 profiling experiments were performed as previously described [26–28].

### Cell viability assays

Cell viability effects were determined using the standard colorimetric MTT reduction assay.

### Xenograft studies

Xenografts were established by injecting 2 × 10^6^ BT-474 cells subcutaneously into ovariectomized 3- to 4-week-old athymic female nude-Doxn1^nu^ mice (Harlan Sprague Dawley, Madison, WI) subcutaneously implanted with slow-release estrogen pellets (Innovative Research) around left forearm using a trocar. Once tumor engraftment was confirmed (>100 mm^3^), mice (*n* = 10 per group) were randomly allocated to each of the following treatment groups: Group A: (i.) Vehicle (PEG-400), (ii.) TVB-3664 (2 mg/kg/day/oral), (iii.) ABT-199 (100 mg/kg/day/oral), and (iv.) TVB-3664 (2 mg/kg/day/oral) + ABT-199 (100 mg/kg/day/oral); group B: (i.) Vehicle (PEG-400), (ii.) TVB-3664 (2 mg/kg/day/oral) alone, (iii.) ABT-263 (100 mg/kg/day/oral) alone, and (iv.) TVB-3664 (2 mg/kg/day/oral) + ABT-263 (100 mg/kg/day/oral). For oral dosing, TVB-3664 was formulated in 100% PEG-400 and diluted with water to a final PEG concentration of 30% immediately before dosing. Tumor volume was calculated by 3D measurements using the formula: tumor volume (mm^3^) = (length × width × height)/2. Tumor volume values (mean ± S.D.) were calculated weekly over a 4 week-period for each experimental group using a Vernier caliper.

### Statistical analysis

At least three independent experiments were performed with *n* ≥ 3 replicate samples per experiment. Data are presented as mean ± S.D. Student’s *t* test was used to compare the means between two groups; comparisons of means of ≥3 groups were performed by one-way ANOVA and Dunnett’s *t*-test for multiple comparisons using GraphPard Prism (GraphPad Software, San Diego, CA). *P*-values <0.05 and <0.005 were considered to be statistically significant (denoted as * and **, respectively). All statistical tests were two-sided.

## Results

### The extent of FASN inhibition-induced apoptotic cell death relates to FASN expression status

**Fig. 2:**
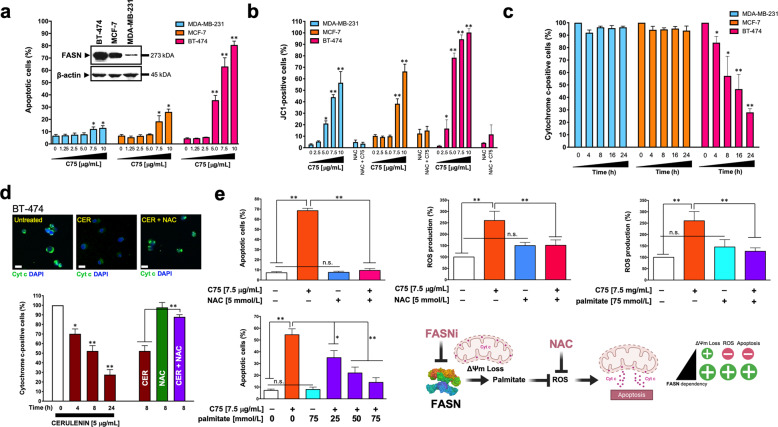
FASN inhibition promotes high levels of apoptotic cell death in FASN-overexpressing breast cancer cells. **a**. BT-474, MCF-7, MDA-MB-231 cells were treated with graded concentrations of C75 or vehicle (DMSO) for 48 h and apoptosis was evaluated by annexin V/propidium iodide staining using two-color flow cytometry. The inset shown the expression of FASN protein in the three cell lines. **b** Flow cytometry assessment of JC-1 fluorescence in cells treated with graded concentrations of C75 or vehicle (DMSO) for 48 h. Alternatively, cells were treated with 7.5 μg/mL C75 in the absence or presence of 5 mmol/L N-acetylcysteine (NAC). **c** Flow cytometry-based assessment of mitochondrial cytochrome c following exposure to C75 (7.5 μg/mL). **d** *Top*. Representative immunofluorescence images of cytochrome c staining in BT-474 cells treated with cerulenin in the absence or presence of N-acetylcysteine (NAC). Scale bar is 10 μm. *Bottom*. Flow cytometry-based assessment of mitochondrial cytochrome c following exposure to cerulenin in the absence or presence of NAC. **e** *Left*. Quantification of apoptosis and reactive oxygen species (ROS) in BT-474 cells treated with C75 in the absence or presence of NAC or palmitate. *Right*. Mitochondrial depolarization appears to be a common response to FASN inhibition irrespective of FASN status; however, the FASNi-driven decrease in ∆ψ_m_ levels appears to have to reach a certain threshold to elicit the release of mitochondrial cytochrome c accompanying apoptotic cell death, which is restricted to FASNi-sensitive cancer cells in an apparently ROS-dependent manner. All data are presented as mean ± SD (*n* = *3*), *p* < 0.05 and *p* < 0.005 (* and **, respectively).

We first examined apoptotic cell death using the semi-synthetic FASNi C75 in three breast cancer cellular models expressing distinct levels of FASN: BT-474 »> MCF-7 > MDA-MB-231 (Fig. 2a, inset). C75 significantly and dose-dependently increased the number of annexin V-positive BT-474 cells relative to vehicle-treated control cells (Fig. 2a). A drastically smaller but still significant increase in annexin V-positive cells was observed in C75-treated MCF-7 and MDA-MB-231 cells. Equivalent findings were found when using the natural FASN inhibitor cerulenin (Fig. S1a).

### FASN inhibition promotes ROS-dependent mitochondrial cytochrome c release during apoptosis

We assessed whether FASN inhibition was accompanied by changes in two apoptosis-related readouts, namely mitochondrial transmembrane potential (∆ψ_m_) and cytochrome c release [29–32]. All three cell lines responded to C75 with a dose-dependent increase in the number of cells positive for JC-1 green fluorescence (Fig. 2b), indicating mitochondrial membrane depolarization. C75-induced loss of ∆ψ_m_ was prevented by N-acetylcysteine (NAC) [33, 34] (Fig. 2b), suggesting a role for ROS in the loss of ∆ψ_m_ upon FASN blockade. ROS production was significantly increased by C75 in BT-474 and MCF-7 cells, but not in MDA-MB-231 cells, as revealed by DCF staining (data not shown).

A time-dependent release of cytochrome c was observed only in FASNis-treated BT-474 cells but not in MCF-7 and MDA-MB-231 cells, as measured by flow cytometry (Fig. 2c, d). Co-treatment with NAC treatment blocked the ability of the FASNi to induce apoptotic cell death (Fig. 2e, Fig. S1b), and prevented ROS production and cytochrome c release in FASNis-sensitive cancer cells (Fig. 2d, e). Co-treatment with the FASN end-product palmitate largely prevented C75-induced ROS production and effectively protected BT-474 cells from apoptosis in a dose-dependent manner (Fig. 2e).

### FASN inhibition activates redox-sensing kinases

As ROS production commonly results from redox stress, and considering that redox-associated NADPH is rapidly consumed by FASN [24], we examined the effects of FASN inhibition on NADPH accumulation. We found a dramatic, dose-dependent accumulation of NADPH in BT-474 cells treated with C75 (Fig. S1c). C75 promoted a more modest increase in the NADPH/NADP^+^ ratio in both MCF-7 and MDA-MB-231 cells (Fig. S1c).

Redox imbalance causes the activation of stress-related proapoptotic kinases such as Jun N-terminal kinase (JNK) and p38 mitogen-activated protein kinase (p38 MAPK) [35, 36]. Although with different activation dynamics that might reflect the induction of stress responses to attenuate ROS-mediated JNK activation, FASNi augmented the phosphorylation status of both JNK and p38 MAPK, which was largely prevented by NAC (Fig. S1d). FASN inhibition also strongly activated AMP-activated protein kinase (AMPK), a key regulator of metabolism and survival during energy stress that senses intracellular redox signals [37], and this was prevented by NAC (Fig. S1d).

### FASN inhibition upregulates pro-death BH3-only proteins

**Fig. 3:**
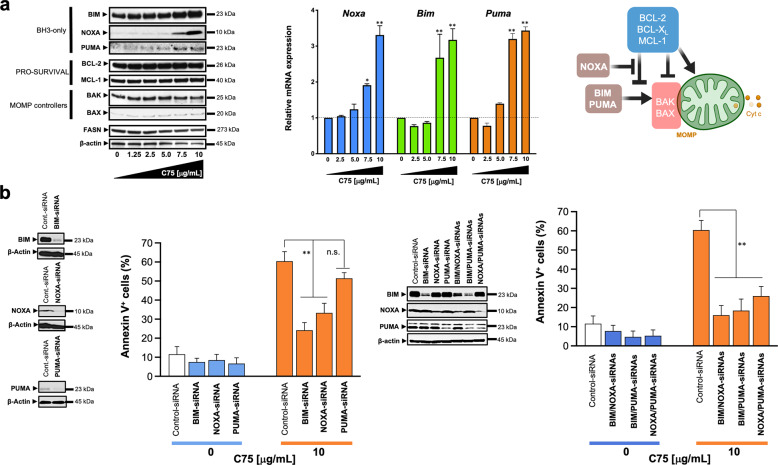
BH3-only proteins mediate FASN inhibition-induced apoptosis. **a** FASN inhibition activates pro-apoptotic BH3-only proteins. *Left*. Representative immunoblot analysis of NOXA, BIM, PUMA, BAK, BAX, BCL-2, MCL-1, and FASN in cell lysates from BT-474 cells treated with graded concentrations of C75 for 48 h. *Right*. Q-PCR analyses of the expression of *Bim*, *Noxa*, and *Puma*. Before RNA extraction, cells were treated with C75 in the presence of the pan caspase inhibitor Q-VD-OPh (5 µmol/L) for 48 h, collected by trypsinization, sedimented at 250×*g* for 10 min and frozen on dry ice. Data are presented as mean ± SD (*n* = *3*), *p* < 0.05 and p < 0.005 (* and **, respectively). **b**. Depletion of BH3-only proteins prevents FASNi-induced apoptotic cell death. Annexin V/propidium iodide staining-based flow cytometric assessment of apoptotic cell death in C75-treated BT-474 cells previously transfected with either control, BIM, NOXA, or PUMA single siRNAs (*top panels*) or their combinations (*bottom panels*). Annexin V^+^ cells data are presented as mean ± SD (*n* = *3*), *p* < 0.05 and *p* < 0.005 (* and **, respectively). Also shown are representative immunoblots (*n* = *3*) of the BIM, NOXA, and PUMA knockdown efficiencies achieved by the corresponding siRNAs.

Using pooled populations of both adherent and dead floating cells, we found that the expression of the multidomain anti-apoptotic proteins BCL-2, BCL-X_L_, and MCL-1 remained mostly unchanged following pharmacological inhibition of FASN activity (Fig. 3a, S1e). C75 treatment resulted in a robust dose-dependent upregulation of the BH3-only BCL2 members BIM, NOXA, and PUMA at both protein and mRNA levels (Fig. 3a). Inhibition of p38 MAPK activity with SB203580 lessened the ability of C75 to upregulate BIM, NOXA, and PUMA (Fig. S1f, g), indicating that ROS-driven activation of stress-induced kinases is linked to the induction of BH3-only proteins in FASN-inhibited cancer cells. The protein synthesis inhibitor cycloheximide blocked the activation of BH3-only proteins in C75-treated cells (Fig. S1f, g), suggesting that their accumulation after FASN inhibition requires de novo protein synthesis.

siRNA-mediated knockdown of BIM or NOXA significantly reduced (up to 60 and 40% reduction, respectively) apoptotic cell death in C75-treated BT-474 cells (Fig. 3b). siRNA-mediated depletion of PUMA had less significant effect on C75-induced apoptosis. Although those combinations containing the BIM-targeted siRNA (BIM/NOXA and BIM/PUMA) were the most effective in preventing C75-induced apoptotic cell death, the dual NOXA/PUMA silencing was also capable of significantly reducing the extent of apoptosis in FASN-inhibited cells (Fig. 3b). Palmitate rescued the downregulation of FASN expression and the upregulation of BIM, NOXA, and PUMA in response to C75 (Fig. S1h).

### FASN status is a cancer cell-intrinsic determinant of mitochondrial priming

**Fig. 4:**
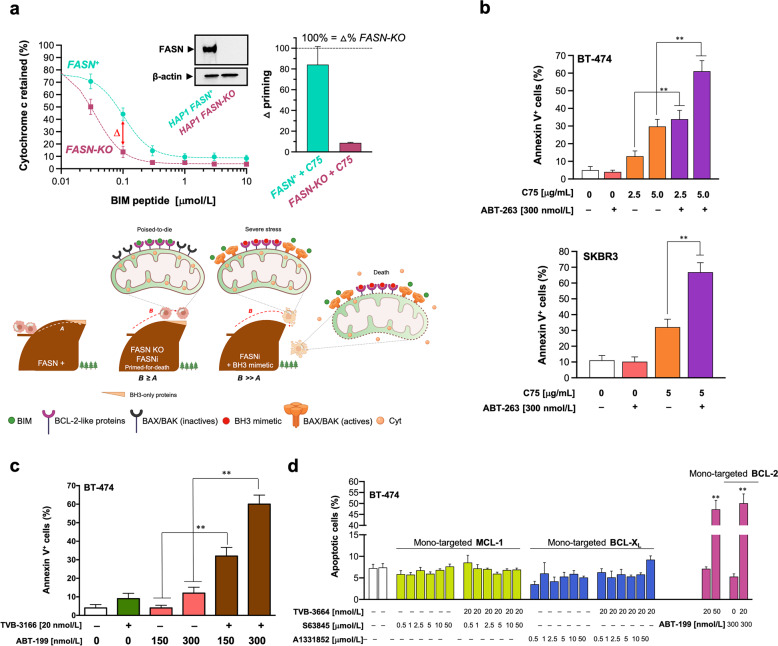
FASN inhibition exacerbates the pro-apoptotic activity of BCL2-targeting BH3 mimetics. **a** FASN is a determinant of mitochondrial priming. *Left*. Dynamic BH3 profiling (DBP) assay of *FASN*-expressing HAP1 parental cells and HAP1 *FASN-KO* isogenic derivatives after treatment with increasing concentrations of the BIM BH3 peptide. Representative immunoblot analysis of FASN in cell lysates from HAP1 *FASN*^*+*^ parental cells and HAP1 *FASN-KO* isogenic derivatives. Results expressed as ∆% priming represents the increase in priming compared to FASN-containing HAP1 control cells. ∆% priming after 16 h incubation with C75 was measured by enhanced BIM-induced cytochrome c release compared to DMSO-treated controls. Data are presented as mean ± SD (*n* = *4*). *Right*. Mitochondrial priming is depicted as proximity to the “cliff’s edge” (i.e., the apoptotic threshold). FASN-expressing cells (FASN + ) are at distance *A* from the edge. FASN suppression/inhibition causes mitochondrial membrane depolarization (an early event in the onset of apoptosis). The distance that FASN inhibition pushes the cells towards the clifftop is represented by the movement *B*, which is sufficient to push the cells to the clifftop (*B* ≥ *A*). This model predicts that the ability of FASN-inhibited cells to cross the apoptotic threshold and fall to the “cell death valley” can be accelerated in the presence of BH3 mimetics, which promote a deepening of the slope towards the valley (*B » A*). Created with BioRender.com. **b**, **c**, **d** Annexin V/propidium iodide staining-based flow cytometric assessment of apoptotic cell death in BT-474 and SKBR3 cells treated with FASNis (C75, TVB-3166, TVB-3664) in the absence or presence of ABT-263, ABT-199, S63845, and A-1331852. Representative experiments showing the percentage of annexin V-positive cells in each experimental condition and quantification of apoptosis are shown. Data are presented as mean ± SD (*n* = *3*), *p* < 0.05 and *p* < 0.005 (* and **, respectively).

FASN activity might regulate cancer cell survival by fine-tuning the mitochondrial threshold for apoptosis, known as “mitochondrial priming” [38–47]. To test this hypothesis, we took advantage of the human near-haploid cell line HAP1 and its isogenic derivative carrying a loss-of-function mutation in *FASN* as a suitable model system for defective de novo FA synthesis [48]. We envisioned that CRISPR/Cas9-mediated specific suppression of FASN might suffice to drive early changes in the BCL-2 family of proteins preceding the apoptotic process engagement, which can be detected by the dynamic BH3 profiling assay [38, 39, 45]. The more “primed” a cell is, the more sensitive its mitochondria will be to the synthetic BIM BH3 peptide, which promiscuously binds to all the anti-apoptotic BCL-2 family members and is a probe of overall mitochondrial priming. The half-maximal concentration of the BIM peptide required to promote cytochrome c release decreased by ~3-fold in *FASN-KO* cells compared to FASN-expressing isogenic counterparts (Fig. 4a, *left*), revealing that the BIM BH3 peptide permeabilized the mitochondria more efficiently upon FASN depletion. Accordingly, *FASN-KO* cells exhibited a Δ% priming of ~23% at 0.1 μmol/L BIM BH3 peptide. An equivalent Δ% priming occurring in HAP1 cells treated with a sub-optimal concentration of the FASNi C75 (5 μg/mL) was largely prevented in *FASN-KO* HAP1 cells (Fig. 4a, *left*).

### FASN inhibition enhances sensitivity to the BCL-2 family inhibitor navitoclax/ABT-263

If FASN suppression elicits an enhanced mitochondrial priming response, then FASNis should overcome resistance to BH3 mimetics (Fig. 4a, *right*). We first questioned whether cancer cells treated with C75 showed increased sensitivity to BH3 mimetic drugs such as the dirty/promiscuous antagonist of BCL-2, BCL-X_L_, and BCL-W navitoclax/ABT-263 [49, 50]. Whereas single-agent navitoclax failed to trigger apoptotic cell death, addition of C75 noticeably synergized (2–3-fold) with navitoclax/ABT-263 (Fig. 4b). This ability involved a synergistic amplification of ROS generation (Fig. S1i). In SKBR-3 cells, which express the highest cellular levels of FASN (up to 28% by weight of the cytosolic proteins) yet described in human established cell lines [26, 27, 51, 52], a clear apoptotic synergism was observed when C75 was added to navitoclax/ABT-263, which had no impact against SKBR-3 cells as single agent (Fig. 4b). The apoptosis-resistant phenotype of low/moderate-FASN expressing MDA-MB-231 and MCF-7 treated with navitoclax/ABT-263 remained unaltered by the addition of C75 (Fig. S1j).

### A clinical grade FASNi sensitizes cancer cells to the BCL-2-specific BH3 mimetic venetoclax/ABT-199 in vitro

We explored the nature of the interaction between the recently developed small-molecule FASNi TVB-3166 [12] and venetoclax/ABT-199, an FDA-approved BH3 mimetic that specifically targets BCL-2 rather than multiple BCL proteins [53–55] (Fig. 4c). HAP1 *FASN-KO* cells were largely refractory to the cytotoxic effects of TVB-3166 (Fig. S1k), thereby demonstrating the FASN-dependent mechanism of action of the TVB series of FASNis. Whereas single-agent venetoclax/ABT-199 failed to induce any significant level of apoptotic cell death, addition of TVB-3166 dramatically enhanced the capacity of venetoclax/ABT-199 to promote apoptotic cell death in BT-474 cells (Fig. 4c).

To definitely delineate the right BH3 mimetic that should accompany a FASNi to work better in a clinical setting [56, 57], we finally examined the nature of the apoptotic interaction between mono-targeted BH3 mimetics and the TVB-3166-related molecule TVB-3664—a close analog of the clinical-grade FASNi TVB-2640 [56–61]. Co-administration of TVB-3664 synergistically augmented the apoptosis-inducing activity of the BCL-2 specific BH3-mimetic venetoclax/ABT-199 (Fig. 4d). TVB-3664, however, failed to sensitize breast cancer cells to BH3-mimetics mono-targeting MCL-1 (S63845) and BCL-X_L_ (A1331852) (Fig. 4d) [62].

### A clinical grade FASNi enhances sensitivity to navitoclax/ABT-263 and venetoclax/ABT-199 in vivo

**Fig. 5:**
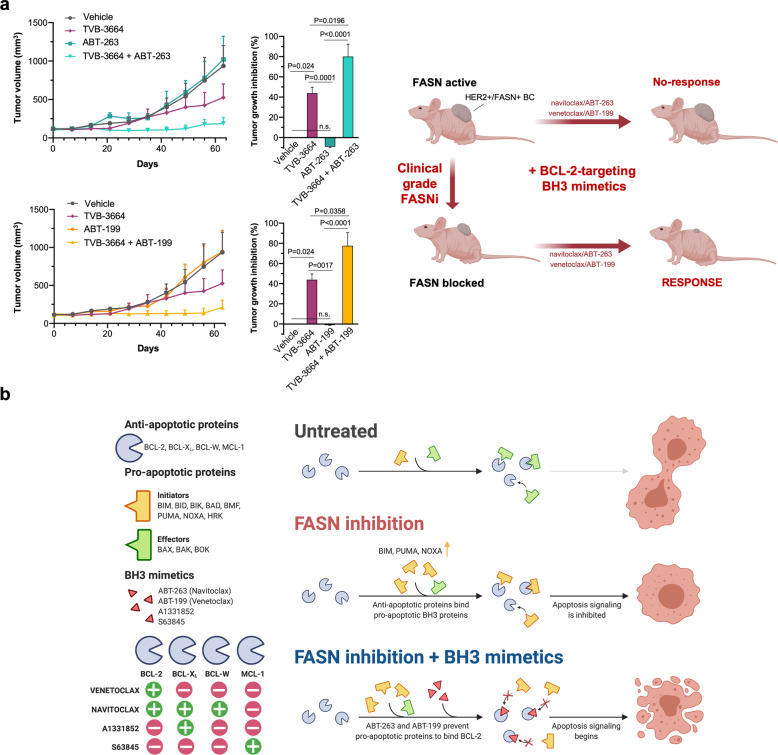
FASN inhibition sensitizes human breast tumor xenografts to BCL-2-targeting BH3 mimetics. **a** *Left*. Growth of BT-474 xenograft tumors in athymic female mice treated with BH3 mimetics navitoclax/ABT-263 (*top*) and venetoclax/ABT-199 (*bottom*) in the absence or presence of the FASNi TVB-3664. The maximum length for each treatment was 63 days. Results are presented as the mean tumor volume ± S.D. (*n* = 10 mice/experimental group). Tumor growth inhibition (TGI) was calculated as the percentage of tumor growth, relative to tumor size at the start of treatment, in drug-treated groups compared to vehicles-treated group. *Right*. In vivo findings from the HER2 + /FASN-overexpressing breast cancer model BT-474 uncovers a novel FASN-dependent mitochondrial priming that links de novo FA biosynthesis to the intrinsic apoptotic threshold in breast cancer cells. The discovery that FASN-inhibited cancer cells exist in an apoptosis-prone state highly sensitive to BCL-2-targeting BH3 mimetics might warrant clinical exploration in patients with HER2 + /FASN-addicted breast carcinomas (see the discussion section). FASN inhibition increases mitochondrial priming and enhances breast cancer cell sensitivity to BCL2-targeting BH3 mimetics: a working model (**b**). FASN-inhibited breast cancer cells exhibit an exacerbated apoptotic sensitivity to BH3 mimetics. In FASN-addicted cancer cells, FASNis promote increased levels of pro-apoptotic activators such as BIM, thereby poising these cells ready for apoptotic cell death. However, FASN-inhibited cells can survive with high mitochondrial priming because they become “addicted” to the expression of anti-apoptotic proteins such as BCL-2 to sequester BIM and ensure survival. Cancer cell death will occur if the sequestration capacity of anti-apoptotic proteins is exceeded by levels of pro-apoptotic proteins, which can be pharmacologically achieved with BH3 mimetic drugs capable of disrupting the ability of anti-apoptotic BCL-2 family members to neutralize BH3-only proteins-driven activating events. Specifically, co-administration of a FASNi synergistically augments the apoptosis-inducing activity of the dual BCL-X_L_/BCL-2 inhibitor ABT-263 (navitoclax) and the BCL-2 specific BH3-mimetic ABT-199 (venetoclax). FASN inhibition, however, fails to sensitize breast cancer cells to MCL-1- and BCL-X_L_-selective inhibitors such as S63845 and A1331852. Adapted from “MCL-1 Inhibition for Leukemia Treatment” by BioRender.com (2021). Retrieved from https://app.biorender.com/biorender-templates.

We finally sought to determine the efficacy of combining navitoclax/ABT-263 or venetoclax/ABT-199 with TVB-3664 against BT-474 human breast cancer xenografts in nude mice. BH3 mimetics and TVB-3664 were administered by oral gavage to mimic human oral drug administration. Both navitoclax/ABT-263 and venetoclax/ABT-199 failed to elicit any tumor growth delay of BT-474 xenograft tumors; notably, single agent TVB-3664 was notably efficacious in producing a tumor response (44% tumor growth inhibition) (Fig. 5a). The completely lack of anti-tumor efficacy of navitoclax/ABT-263 and venetoclax/ABT-199 as single agents was fully circumvented when FASN activity was pharmacologically targeted in BT-474 tumor xenografts; thus, when administered in combination with the FASNi TVB-3664, navitoclax/ABT-263 and venetoclax/ABT-199 caused strong tumor growth inhibition (80% and 78%, respectively; Fig. 5a). Combination therapy appeared to be well-tolerated, with mice maintaining normal body weight (Fig. S1k).

## Discussion

A major roadblock to the clinical application of FASNis is that the mechanism behind the resistance versus sensitivity of cancer cells to death signaling triggered by FASN blockade remained poorly understood [11–14]. We here show the ability of FASN activity to dictate how close a cell is to the threshold of apoptosis, a property called mitochondrial priming [38–48]. Mitochondria in FASN-inhibited cancer cells exist in a primed-for-death state that offers a novel therapeutic opportunity to treat breast cancer by inducing hypersensitization to pro-apoptotic BH3 mimetic drugs (Fig. 5b).

The fate of cancer cells exposed to FASNis might depend on their proximity to an apoptotic threshold. Elevated levels of BCL-2-like pro-survival proteins bound to pro-apoptotic family members (e.g., BIM) appear to prepare cancer cells for apoptosis following FASNi. Cell death induced by FASNis is diminished by concurrent loss of pro-apoptotic proteins, suggesting that FASN activity regulates cancer cell survival by fine-tuning the mitochondrial threshold for apoptosis. Thus, while FASNis may not overtly affect cancer cell viability, they heighten mitochondrial priming, which shifts cancer cells towards a primed-for-death state that is addicted to anti-apoptotic proteins to ensure survival but allows a BH3-mimetic to unleash the mitochondrial pathway of apoptosis. Specifically, counteracting the binding of pro-apoptotic proteins with BH3-mimetics antagonizing BCL-2 (navitoclax and venetoclax) rather than MCL-1 (S63845) and/or BCL-X_L_ (A1331852) abolishes this protected state (Fig. 5b), leading to increased apoptosis in FASN-inhibited cancer cells.

BCL-2-targeting BH3 mimetics alone have shown promising activity in hematological malignancies, but they have been mostly ineffective for solid tumors [63]. The response to BH3 mimetics requires the alteration of the proapoptotic/antiapoptotic balance of BCL-2 members through concomitant exposure to chemotherapy, hormone therapy, or mTOR inhibitors [64–66]. Here, FASNi-promoted upregulation of pro-apoptotic BH3-only proteins was rescued by the FASN end-product palmitate, revealing that starvation of endogenously produced FAs should be added to the list of metabolic stresses capable of eliciting mitochondrial priming and augmented responsiveness to BH3 mimetics [67–71]. Regardless of the interconnected hierarchical model explaining how the BCL-2 family proteins dictate cellular survival versus mitochondrion-dependent cell death [72–76], we now provide solid evidence that intrinsic/primary unresponsiveness of cancer cells to BCL2-targeting BH3 mimetics can be circumvented by promoting a high mitochondrial primed-for-death state with FASNis.

The in vivo efficacy data in a xenograft cancer model validating the actionable synergistic interaction between a clinical grade-like FASNi and the BH3 mimetics ABT-263/navitoclax and venetoclax/ABT-199 strongly support the exploration of their combination in the clinic. Transcriptomic and proteomic data have confirmed that HER2^+^ breast tumors are the highest FASN-expressors among breast cancer subtypes [77–81]. The HER2-FASN lipogenic axis therefore delineates a subgroup of patients that might benefit from therapeutic regimens containing FASNis and BCL-2 BH3 mimetics. The recent discovery that brain metastases in HER2^+^ breast cancer patients rely on FASN [82, 83] illuminates the important translational impact of combining blood–brain–barrier penetrable versions of FASNis and BCL-2 BH3 mimetics such as venetoclax, which has already shown a potential efficacy in the treatment of malignancies with central nervous system involvement [84–86].

## Conclusions

We here add FASN-dependent endogenous lipogenesis to the list of metabolic pathways closely intertwined with apoptotic cell death in cancer cells. We propose that heightened mitochondrial priming is the basis for the apoptotic hypersensitivity of cancer cells starved of FASN-synthesized de novo FAs. Thus, the capacity of FASN activity to regulate mitochondrial priming correlates with, and may be a determinant of the therapeutic index of FASNis. Our findings support a mitochondrial basis to explore the actionable synergistic interaction between FASNis and BH3-mimetics targeting BCL-2 in the clinic setting.

## Supplementary information

Supplementary information

## Data Availability

The data that support the findings of this study are available from the corresponding authors, upon reasonable request.
