# Supplementary material for: Fatty acid synthase (FASN) regulates the mitochondrial priming of cancer cells
Source: Cell Death Dis. 2021 Oct 21;12(11):977. doi: 10.1038/s41419-021-04262-x (PMC8531299; doi:10.1038/s41419-021-04262-x)
Supplement: Supplementary file 1 — Supplementary information [file 41419_2021_4262_MOESM1_ESM.docx]

**Fatty acid synthase (FASN) regulates**

**the mitochondrial priming of cancer cells**

**–SUPPLEMENTARY INFORMATION–**

**
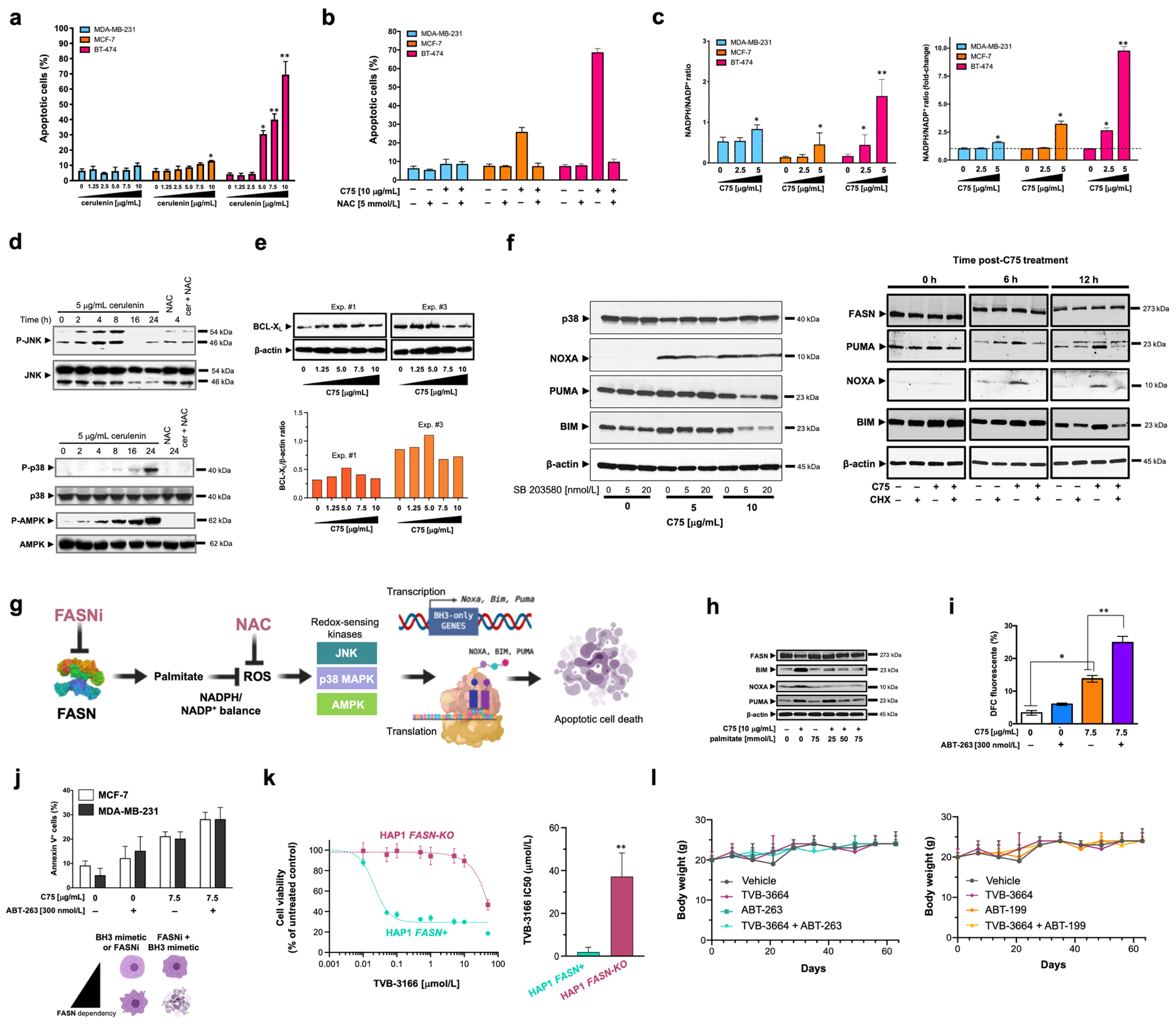
**

**Figure S1. a.** BT-474, MCF-7, MDA-MB-231 cells were treated with graded concentrations of cerulenin or vehicle (DMSO) for 48 h and apoptosis was evaluated by annexin V/propidium iodide staining using two-color flow cytometry. **b** Quantification of apoptosis in BT-474, MCF-7, and MDA-MB-231 cells treated with C75 in the absence or presence of NAC or palmitate. **c.** NADPH/NADP^+^ ratio (*left*) and fold-change in NADPH/NADP^+^ ratio (*right*) in BT-474, MCF-7, and MDA-MB-231 cells cultured in the absence or presence of graded concentrations of C75 for 48 h. Data are presented as mean ± SD (*n=3*), p < 0.05 and p < 0.005 (* and **, respectively). **d.** Immunoblotting assessment of the activating phosphorylation of p38-MAPK, JNK, and AMPK in BT-474 cells treated with cerulenin in the absence or presence of N-acetylcysteine (NAC). **e.** Representative immunoblotting assessments of BCL-X_L_ expression in BT-474 cells treated with graded concentrations of C75. **f.** Representative immunoblotting analyses (*n=3*) of BH3-only proteins NOXA, PUMA, and BIM in the absence or presence of the p38 MAPK inhibitor SB203580 (*left panels*) and the protein synthesis inhibitor cycloheximide (CHX; *right panels*). **g.** In FASN-addicted breast cancer cells, FASN inhibitor (FASNi) suppresses the critical role of FASN in maintaining cellular redox homeostasis and its inhibition leads to NADPH accumulation, reactive oxygen species (ROS) production and, consequently, activation of stress-associated/redox-sensing kinases (JNK, p38 MAPK, AMPK). FASNi triggers the up-regulation of several members of the BH3-only group of BCL-2 proteins (BIM, PUMA, and NOXA). Each step of the FASNi/BH3-only protein pathway can be blocked with a ROS scavenger or the FASN end-product palmitate, thus confirming that the delineated pro-apoptotic cascade reflects on-target effects of FASNi. Although several studies have implicated JNK, p38 MAPK, and AMPK as direct/indirect contributors to the transcriptional activation of *Bim*, *Puma*, and *Noxa* [1-5], our current data does not unambiguously clarify if stress-associated/redox-sensing kinases are causal mediators of FASNi-induced activation of the BH3-only proteins BIM, PUMA, and NOXA at the transcriptional level and, therefore, further studies are needed to clarify this framework. **h.** Representative immunoblotting analysis of FASN, BIM, PUMA, and NOXA in cell lysates from BT-474 cells treated with C75 in the absence or presence of palmitate. **i.** Quantification of reactive oxygen species (ROS) measured by DCFH-DA oxidation in BT-474 cells treated with C75 in the absence or presence of ABT-263. Data are presented as mean ± SD (*n=3*), p < 0.05 and p < 0.005 (* and **, respectively). **j.** Annexin V/propidium iodide staining-based flow cytometric assessment of apoptotic cell death in MCF-7 and MDA-MB-231 cells treated with ABT-263 in the absence or presence of C75. Data are presented as mean ± SD (*n=3*). **k.** *Left.* A representative dose-response curve of TVB-3166 in HAP1 FASN^+^ and HAP1 *FASN-KO* derivatives. *Right.* Half-maximal inhibitory concentration (IC_50_) values of TVB-3166 in FASN+ HAP1 and HAP1 *FASN-KO* derivatives. Data are presented as mean (*columns*) ± SD (*bars*) (n=4). p < 0.005 (**). **l.** Mean relative body weights ± S.D. of animals across treatment groups.

**Supplementary information references**

1. Lu J, Quearry B, Harada H. [p38-MAP kinase activation followed by BIM induction is essential for glucocorticoid-induced apoptosis in lymphoblastic leukemia cells.](https://pubmed.ncbi.nlm.nih.gov/16730715/) FEBS Lett. 2006;580:3539-44.
2. Cuadrado A, Lafarga V, Cheung PC, Dolado I, Llanos S, Cohen P, et al. [A new p38 MAP kinase-regulated transcriptional coactivator that stimulates p53-dependent apoptosis.](https://pubmed.ncbi.nlm.nih.gov/17380123/) EMBO J. 2007;26:2115-26.
3. Cazanave SC, Mott JL, Elmi NA, Bronk SF, Werneburg NW, Akazawa Y, et al. [JNK1-dependent PUMA expression contributes to hepatocyte lipoapoptosis.](https://pubmed.ncbi.nlm.nih.gov/19638343/) J Biol Chem. 2009;284:26591-602.
4. [Concannon CG, Tuffy LP, Weisová P, Bonner HP, Dávila D, Bonner C, et al. AMP kinase-mediated activation of the BH3-only protein Bim couples energy depletion to stress-induced apoptosis.](https://pubmed.ncbi.nlm.nih.gov/20351066/) J Cell Biol. 2010;189:83-94.
5. Girnius N, Davis RJ. [JNK Promotes Epithelial Cell Anoikis by Transcriptional and Post-translational Regulation of BH3-Only Proteins.](https://pubmed.ncbi.nlm.nih.gov/29141222/) Cell Rep. 2017;21:1910-1921.
